# Comparative Effectiveness of Balloon Aortic Valvuloplasty via Transradial and Transfemoral Access

**DOI:** 10.1016/j.jscai.2025.104015

**Published:** 2025-11-18

**Authors:** Jonathan X. Fang, Pedro A. Villablanca, Tiberio M. Frisoli, Pedro Engel Gonzalez, James C. Lee, Georgi K. Fram, Leo Kar Lok Lai, Gennaro Giustino, Hussayn Alrayes, Louie B. Kamel-Abusalha, Rama Ellauzi, Samuel Gregerson, Michael Chiang, Kent Chak-yu So, Dee Dee Wang, William W. O’Neill, Brian P. O’Neill

**Affiliations:** a Center for Structural Heart Disease, Henry Ford Health System, Detroit, Michigan; b Department of Cardiology, National Heart Centre Singapore, Singapore; c Department of Medicine and Therapeutics, Prince of Wales Hospital, Chinese University of Hong Kong, Hong Kong SAR, China; d Gagnon Cardiovascular Institute, Morristown Medical Center, Atlantic Health System, Morristown, New Jersey; e College of Medicine and Life Sciences, University of Toledo, Toledo, Ohio; f Department of Medicine, Queen Elizabeth Hospital, Hong Kong SAR, China; g NCH Rooney Heart Institute, Naples, Florida

**Keywords:** balloon aortic valvuloplasty, radial access, severe aortic stenosis

## Abstract

**Background:**

Balloon aortic valvuloplasty (BAV) is commonly performed as a bridge to therapy, for stratification, or as a palliative procedure in cases of severe aortic stenosis. The complication rate of transfemoral access BAV (transfemoral valvuloplasty [TFV]) is comparable to that of transcatheter aortic valve replacement. Transradial access BAV (transradial valvuloplasty [TRV]) is technically feasible; however, comparative data for TFV are lacking. We aim to compare TFV and TRV in terms of technical and hemodynamic success, periprocedural safety, and short-term clinical outcomes.

**Methods:**

Consecutive patients undergoing BAV at a tertiary center from 2021 to 2024 were assessed. TRV was performed with ultrasound guidance and an 8F short sheath equipped with compatible balloons. Hemodynamic success was defined as a reduction in gradient of 30% or more. The primary outcome was the periprocedural composite of a Valve Academic Research Consortium (VARC) 3 major vascular complication, grade 3 to 4 bleeding, and balloon entrapment, and nonaccess-related events, including complete heart block, periprocedural stroke, hypotension, severe aortic insufficiency, and periprocedural death. The secondary outcome was the 30-day composite of all-cause mortality, cardiac-related hospitalization, and discharge failure. Inverse probability of treatment weighting, followed by multivariate regression, was employed to address confounders.

**Results:**

105 TRV and 148 TFV were included. Technical success rate was 96.2% for TRV and 98.7% for TFV (*P* = .21). The primary outcome event rate was significantly lower in the TRV compared to the TFV group: 2.53% vs 17.47%; adjusted odds ratio, 0.13; 95% CI, 0.04-0.49; *P* = .003. Technical and hemodynamic success and secondary outcomes were comparable between TRV and TFV.

**Conclusions:**

In comparison to TFV, TRV is associated with lower rates of periprocedural safety events while maintaining similar short-term clinical outcomes and hemodynamic performance.

## Introduction

Transcatheter aortic valve replacement (TAVR) has transformed patient care for those with severe aortic stenosis (AS). However, assessing suitability for TAVR can be challenging in acutely ill patients with high comorbidity burdens. Moreover, emergency TAVR has a high mortality rate.^1^ Balloon aortic valvuloplasty (BAV) has been used as a temporizing procedure to TAVR in unstable patients, as a stratifying procedure for those with uncertain benefits from TAVR, or as a palliative option for patients unsuitable for TAVR. Nevertheless, BAV has a complication rate that is comparable to that of TAVR when performed via the transfemoral access, primarily due to a high rate of vascular complications.^2^, ^3^, ^4^, ^5^ Therefore, there is a clinical need to develop safer BAV techniques, such as the use of radial access.^6^^,^^7^ Selected balloons can be delivered through adequately sized radial arteries. In a European single-arm study, transradial BAV had a low rate of vascular and bleeding events.^8^^,^^9^ Moreover, performing BAV transradially can also preserve the femoral access for later use, such as for TAVR. To date, there are no comparative data between transradial and transfemoral BAV. We aim to investigate the comparative effectiveness of BAV via transradial and transfemoral access in terms of technique and hemodynamic success, periprocedural safety, and short-term clinical outcomes.

## Materials and methods

### Study population

Consecutive patients presenting for BAV without simultaneous TAVR at a high-volume tertiary center in the United States (Henry Ford Hospital, Detroit, Michigan) from June 2021 to August 2024 were evaluated. Exclusion criteria included BAV as a treatment for paravalvular leak, usage of the biradial mini-BAV technique using 2 peripheral balloons, axillary artery access BAV, treatment with valvular lithotripsy alone, and concurrent procedures favoring femoral access, such as planned mechanical circulatory support and concurrent mitral or tricuspid valve interventions. Severe aortic valve stenosis (AS) was defined as an aortic valve (AV) area of less than 1.0 cm^2^, measured by echocardiogram or cardiac catheterization, and was further categorized into subtypes according to current definitions.^10^

### BAV procedure

A technical procedural guide and a table of balloon compatibility for radial access are provided in Supplemental Appendix S1 and Supplemental Table S1.

**Figure 1:**
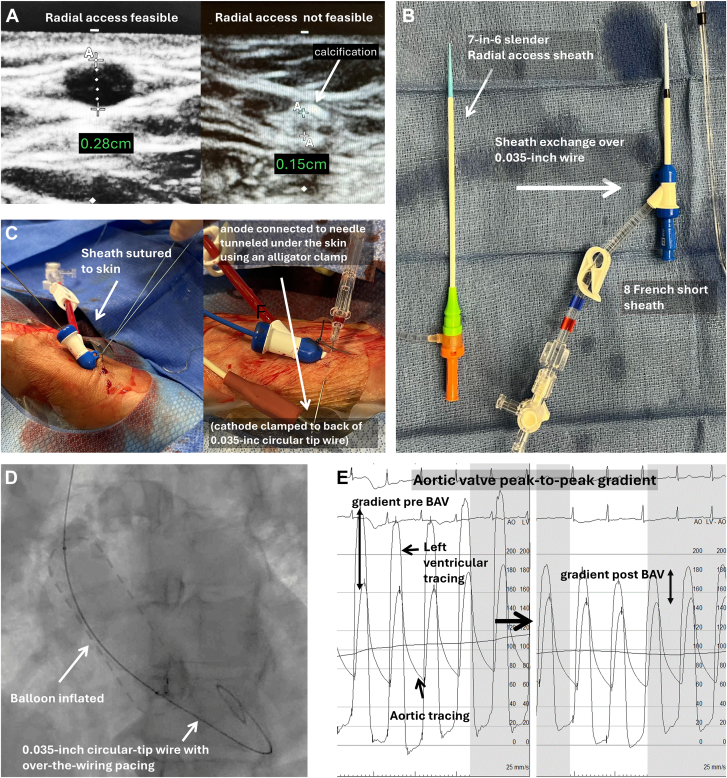
**The balloon aortic valvuloplasty (BAV) procedure.** (**A**) A radial artery with a diameter of 2.8 mm was favorable for access, whereas one with calcification and a diameter of 0.15 cm was not. (**B**) The slender 7F-in-6F radial sheath was first inserted and then upsized to an 8F short sheath over a 0.035-inch wire. (**C**) The 8F short sheath is sutured to the skin. Rapid pacing is performed using over-the-wire pacing, connecting with alligator clamps, with the anode connected to a needle tunneled under the skin and the cathode connected to the back of the 0.035-inch wire. (**D**) Inflation of the balloon valvuloplasty balloon occurs under rapid pacing. (**E**) The pressure gradient across the aortic valve is measured before and after BAV.

In both transradial and transfemoral approaches, ultrasound guidance was mandatory. Transfemoral access was performed with a micropuncture technique integrating both ultrasound and fluoroscopy guidance. For transradial BAV, radial access was achieved with ultrasound guidance using a 7-in-6F Slender radial sheath, followed by upsizing to an 8F short sheath (Merit Medical) over a 0.035-inch J-tip wire. The short sheath was sutured to the skin. Access was deemed feasible based on the a criteria of a minimum radial artery diameter of 2.5 mm and the absence of circumferential calcification on ultrasound (Figure 1A, B). Operators could use the ulnar artery instead of the radial artery if it was considered more favorable based on the same ultrasound criteria. BAV was performed with balloons compatible with 8F sheath access, as determined by bench testing (Table 1). Rapid pacing was performed preferably using the over-the-wire technique (Figure 1C) to facilitate valvuloplasty (Figure 1D), followed by measurement of gradient via a dual-lumen pigtail catheter pre-and-post valvuloplasty (Figure 1E). Access closure was performed using a single pressure wristband (TR Band, Terumo) in the transradial group, with additional wristbands available in case of closure device failure. In the transfemoral group, access closure was performed using suture-based closure devices (Perclose ProGlide, Abbott) as the first option. Radial artery patency was assessed with ultrasound on patients subsequently presenting for TAVR or interventional or diagnostic catheterization procedures. An ultrasound access feasibility period from October 2023 to April 2024 was nested within the cohort, during which all patients presenting for BAV underwent an ultrasound of the radial artery intended for a radial-first approach.

**Table 1:** Baseline characteristics after adjustment by inverse probability of treatment weighting

	Weighted femoral (n = 269.30)	Weighted radial (n = 222.03)	SMD weighted
Age, y	77.65	78.29	–0.06
Male sex	60.72%	59.24%	–0.03
Height, m	1.68 ± 0.01	1.67 ± 0.03	–0.04
Body weight, kg	80.9 ± 2.12	80.1 ± 2.12	0.03
Race			
Caucasian	77.43%	77.04%	–0.01
African American	10.28%	14.15%	0.11
Hispanic	7.83%	4.14%	–0.16
South Asian	2.29%	0.00%	–0.21
East Asian	0.40%	0.00%	–0.09
West Asian	0.00%	4.67%	0.31
Others	1.77%	0.00%	–0.19
Hypertension	91.18%	93.11%	0.07
Hyperlipidemia	56.65%	64.64%	0.16
Diabetes	46.73%	48.95%	–0.04
Smoker	40.00%	46.01%	0.12
Heart failure	56.55%	59.45%	0.06
Atrial fibrillation	44.22%	45.45%	0.02
Mitral valve disease	46.34%	36.67%	–0.196
Tricuspid valve disease	48.99%	45.97%	–0.06
Coronary artery disease	59.86%	57.34%	–0.05
Chronic kidney disease	53.88%	58.29%	0.09
Dialysis	16.82%	21.51%	0.12
Peripheral artery disease	22.86%	25.24%	0.06
COPD/lung disease	30.67%	25.67%	–0.11
Pacemaker/ICD	10.33%	12.34%	0.06
Malignancy	17.23%	17.17%	–0.002
Dementia	1.74%	2.88%	0.08
GFR, mL/min/1.73 m^2^	62.52	55.56	–0.21
Hemoglobin, g/L	10.79	10.84	0.02
Creatinine, mg/dL	1.96	2.19	0.10
Prior ACS	23.27%	21.25%	–0.05
Prior stroke	14.44%	17.05%	0.07
Prior PCI	34.77%	30.75%	–0.09
Prior CABG	7.66%	7.91%	0.01
Risk assessment			
Emergency admission	63.69%	65.65%	0.04
ADHF	66.81%	66.36%	–0.01
Cardiogenic shock	13.50%	14.19%	0.018
STS predicted risk of operative mortality, %	9.79	8.96	0.09
Frailty index	1.56	1.59	0.03
KCCQ score	37.07	38.94	0.11
Electrocardiogram			
Normal QRS complex	66.73%	58.41%	–0.02
LBBB	11.46%	10.76%	–0.02
RBBB	11.61%	21.04%	0.25
Pacing rhythm	10.20%	9.79%	–0.02
Antithrombotic medications			
Aspirin	63.75%	59.6%	–0.09
Clopidogrel	19.94%	18.71%	–0.03
DOAC	28.08%	29.34%	0.02
Warfarin	2.90%	3.33%	0.02
Anatomical characteristics			
LVEF, %	51.49	51.97	0.09
AV mean gradient, mm Hg	32.53	33.88	0.07
AV area, cm^2^	0.85	0.76	–0.196
AI severity			
None/trivial	57.12%	48.26%	–0.03
Mild	34.63%	34.73%	0.03
Moderate	7.14%	14.72%	0.20
Severe	1.11%	2.29%	0.05
Valve type			
Trileaflet	87.08%	87.83%	0.08
Bicuspid	9.00%	9.80%	–0.03
Prosthetic	3.92%	2.38%	–0.11
AS type			
High gradient	43.95%	48.19%	0.07
Low-flow low-gradient	20.90%	16.69%	–0.16
Paradoxical low-flow low-gradient	24.25%	25.36%	0.10
Normal flow low-gradient	10.90%	9.76%	–0.05
LV end diastolic pressure, mm Hg	19.47	18.73	–0.14
LVOT diameter, cm	2.13	2.10	–0.05
RV dysfunction	28.45%	31.24%	–0.09
Indication			
Bridge to TAVR/SAVR	65.65%	69.00%	
Palliative	1.36%	2.98%	0.11
Stratifying	9.00%	12.27%	0.11
Urgent treatment during off-hours	24.02%	15.75%	–20.74

ACS, acute coronary syndrome; ADHF, acute decompensated heart failure; AI, aortic insufficiency; AV, aortic valve; CABG, coronary artery-bypass grafting; COPD, chronic obstructive pulmonary disease; DOAC, direct oral anticoagulant; GFR, glomerular filtration rate; ICD, implantable cardioverter-defibrillator; KCCQ, Kansas City Cardiomyopathy Questionnaire; LBBB, left bundle branch block; LV, left ventricular; LVEF, left ventricular ejection fraction; LVOT, left ventricular outflow tract; PCI, percutaneous coronary intervention; QRS, QRS complex on electrocardiogram; RBBB, right bundle branch block; RV, right ventricular; SAVR, surgical aortic valve replacement; SMD, standardized mean difference; STS, Society of Thoracic Surgeons; TAVR, transcatheter aortic valve replacement.

### Technical success and hemodynamic performance

Technical success was defined as the successful delivery and inflation of a balloon across the AV and the retrieval of the balloon without intraprocedural mortality. Hemodynamic success was defined as a decrease in the peak-to-peak catheterization gradient or echocardiographic mean gradient by 30%, without inducing severe aortic insufficiency (AI), based on currently accepted definitions.^8^^,^^9^ An alternative definition for hemodynamic success in low-gradient AS, defined as an increase in AV area by 30% or an increase in AV area to more than 1 cm^2^, was also explored. To study the relationship between balloon sizing and left ventricular outflow tract (LVOT) area, the relationship between changes in the peak transcatheter peak-to-peak gradient and the echocardiographically measured LVOT diameter was visualized with a scatter plot featuring a best-fit line and 95% CIs for both transradial and transfemoral access groups without extrapolation. Other procedural characteristics, including procedural time, fluoroscopy time, contrast volume, radiation dose, procedural challenges, and vascular closure failure rate, were also compared.

### Clinical outcomes

The primary outcome was the composite safety end point of the procedure, categorized into 3 groups: access-related events, nonaccess-related events, and periprocedural death. Access-related events encompassed major vascular complications according to the Valve Academic Research Consortium (VARC) 3 criteria,^11^ grade 3 to 4 bleeding events based on these criteria, and balloon entrapment. Nonaccess-related events included periprocedural stroke occurring within 24 hours postprocedure, complete heart block, severe AI, and periprocedural hypotension, defined as a decrease in systolic blood pressure to less than 90 mm Hg during or immediately after the procedure, necessitating treatment with vasopressors or mechanical circulatory support. Periprocedural mortality was defined as death within 24 hours of the procedure, including intraprocedural mortality. The secondary outcome was a 30-day time-to-event composite of all-cause mortality, stroke, heart failure, cardiac-related hospitalization, and failure to be discharged by day 30 postprocedure.

### Statistical analysis

Dichotomous variables were presented as percentages and frequencies and compared using the χ^2^ test. Normality of distribution was assessed with the Shapiro-Wilk test. Continuous variables were compared with 2-sample *t* test or the Wilcoxon rank-sum test as appropriate. A propensity score was generated by fitting a logistic regression model with radial access as the dependent variable and including 43 predictors—23 demographic characteristics and 20 clinical assessment and investigation variables (Supplemental Tables S2 and S3 and Supplemental Figures S1 and S2). Inverse probability of treatment weighting (IPTW) was employed to balance the distribution of covariates between the radial and femoral groups and counterfactual inference. Absolute standardized mean difference (SMD) was used to assess between-group imbalances in baseline and weighted populations, with an SMD of <0.2 indicating a well balanced covariate. Covariates with SMD ≥0.2 were further adjusted with weighted multivariate regression models. Kaplan-Meier estimates and log-rank test were used for time-to-event analysis. Patients were censored on the date they received TAVR or surgical AV replacement. An intention-to-treat analysis was conducted to include patients with crossover to femoral access due to equipment delivery failure in the transradial group. Procedural success and hemodynamic outcomes were assessed in the baseline population, whereas primary and secondary clinical outcomes were assessed in the IPTW-adjusted population. A 2-sided *P* value of <.05 was considered statistically significant. All statistical analyses were performed using Stata 18SE (StataCorp LLC).

### Effect modification and sensitivity analysis

Effect modification on the clinical outcomes was tested for subgroups of patients with concurrent percutaneous coronary intervention, stage III or higher chronic kidney disease, peripheral artery disease, cardiogenic shock, acute decompensated heart failure, and bicuspid AV. Multiple sensitivity analyses were conducted, including propensity score matching, restriction to patients without concurrent procedures, redefining periprocedural stroke as an access-related event, redefining analysis time from time of discharge rather than time from procedure, and the use of competing-risk models (Supplemental Tables S4-S6).

### Ethics

Henry Ford Hospital's institutional review board approval was obtained. The ethical principles of the Declaration of Helsinki were followed.

## Results

### Demographic characteristics

**Figure 2:**
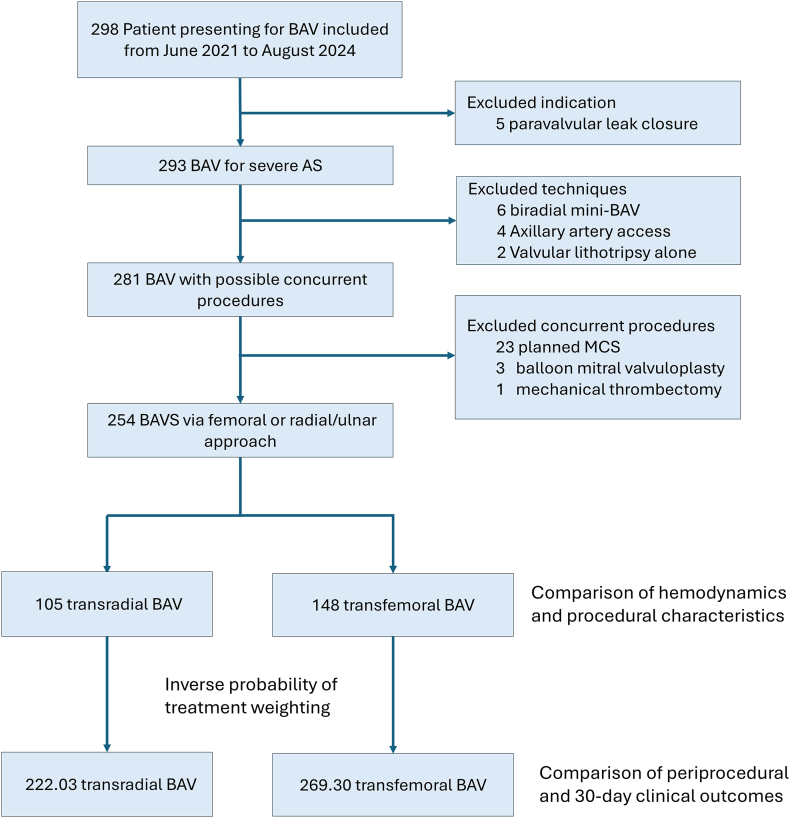
**Study design**. A total of 298 patients with balloon aortic valvuloplasty (BAV) were assessed. After applying the exclusion criteria, 105 patients were included in the radial group and 148 patients in the femoral group. Inverse probability of treatment weighting was utilized to address confounders. Hemodynamic performance and procedural characteristics were compared within the baseline population. Periprocedural and 30-day clinical outcomes were compared in the inverse probability of treatment weighting–adjusted population. MCS, mechanical circulatory support.

One hundred five BAV via the radial approach and 148 via the femoral approach were included for analysis after excluding 44 patients (Figure 2). The transradial group also included 6 patients who had BAV performed through ulnar access based on operator preference. Baseline characteristics in the IPTW population are summarized in Table 1. After IPTW, covariates between the transradial and transfemoral groups were well balanced, achieving an SMD of <0.2 in most covariates, with exceptions including glomerular filtration rate (SMD, –0.21), South Asian ethnicity (SMD, –0.21), moderate preexisting AI (SMD, 0.20), underlying right bundle branch block (SMD, 0.25), and emergency BAV during off-hours without prior heart team evaluation (SMD, –0.21) (Table 1). These covariates were additionally adjusted for using multivariate models.

### Technical and hemodynamic success

Technical success was achieved in 101 out of 105 patients (96.2%) in the transradial group and 146 of 148 patients (98.6%) in the transfemoral group (*P* = .21) (Central Illustration). Crossover to femoral occurred in 4 patients (3.8%) in the transradial group due to the inability to pass the balloon (n = 1) or the sheath (n = 1), or due to radial artery spasm (n = 2). Three of these 4 patients had end-stage kidney disease and were on hemodialysis via an arteriovenous fistula in the contralateral arm. One patient in the transradial group experienced balloon entrapment, which was managed without vascular trauma by cutting the balloon's shaft, followed by snare removal via femoral access. In the femoral group, one patient had balloon delivery failure due to diffuse peripheral arterial disease, and another patient developed a concurrent iliac artery laceration along with cardiac perforation and tamponade, resulting in intraprocedural death. Thirty-six patients were evaluated with ultrasound during the radial-first access feasibility period, with the radial access deemed favorable for access on USG in 30 (83.33%) patients and unfavorable in 6 (16.66%) patients based on the prespecified ultrasound criteria. Of note, 4 out of 6 patients with unfavorable ultrasound findings had end-stage kidney disease.

**Central Illustration:**
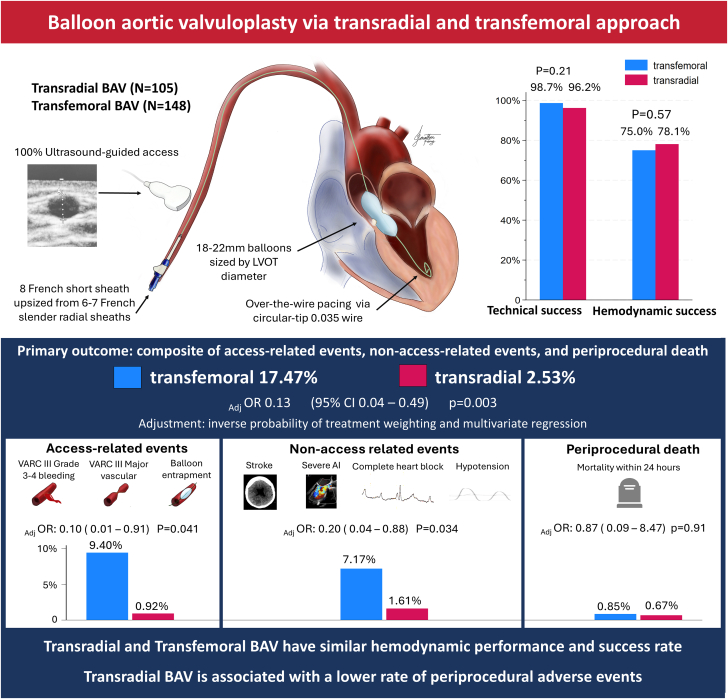
**The primary outcome was a composite of access-related events, nonaccess-related events, and periprocedural death.** Access-related events included Valve Academic Research Consortium (VARC) 3 major vascular complications, VARC 3 grade 3 to 4 bleeding, and balloon entrapment. Nonaccess-related events included periprocedural stroke, severe aortic regurgitation, complete heart block (third degree atrioventricular block), and hypotension. The comparison was made in the inverse probability of treatment weighting–adjusted population using multivariate logistic regression. The adjusted odds ratios (_adj_OR), 95% CI, and *P* values are shown for the composite primary outcome as well as for access-related events, nonaccess-related events, and periprocedural death. There were overall significantly lower rates of access-related and nonaccess-related events, as well as the overall composite outcome. Technical success rate and hemodynamic success rate were comparable. BAV, balloon aortic valvuloplasty; LVOT, left ventricular outflow tract.

**Figure 3:**
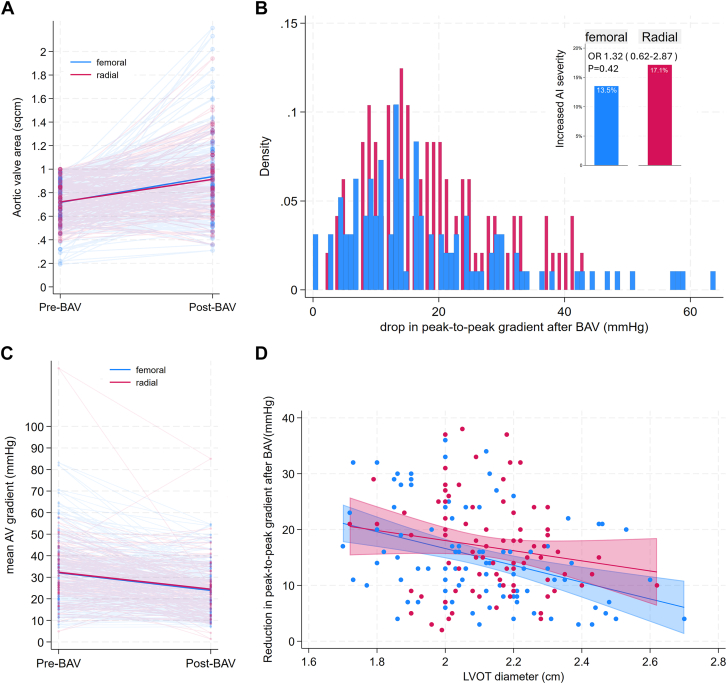
**Hemodynamic results.** (**A**) A connected scatter plot of aortic valve (AV) area measured via echocardiogram before and after balloon aortic valvuloplasty (BAV) in the transradial and transfemoral groups exhibits good overlap with no significant overall difference. (**B**) A histogram depicting the drop in peak-to-peak catheter gradient after BAV in the transradial and transfemoral groups shows an overall similar pattern, despite a few outliers in the transfemoral group. Top right corner: a bar chart illustrating the percentage of cases with increased aortic insufficiency (AI) after BAV in the transradial and transfemoral groups reveals no significant difference in frequency, with an odds ratio of 1.32 (95% CI, 0.62-2.87), *P* = .42. (**C**) A connected scatter plot of the mean AV gradient measured via echocardiogram before and after BAV in the transradial and transfemoral groups demonstrates overall similar results in both groups. (**D**) A scatter plot with linear regression-fitted values and a 95% CI representing the reduction in peak-to-peak catheterization gradient after BAV against the left ventricular outflow tract (LVOT) diameter measured via echocardiogram in the transradial and transfemoral groups shows an overall similar trend in both radial and femoral groups, with a more pronounced drop in peak-to-peak gradient toward LVOT diameter. No extrapolation was performed.

Hemodynamic success was similar in 75.0% of the femoral group and 78.1% of the radial group, with numerically and statistically similar changes in invasively and echocardiographically measured gradients and valve areas, all with *P* > .05 (Table 2 and Figure 3A-C). Despite the conservative balloon sizing in the transradial group, there was no difference in hemodynamic performance regardless of LVOT area (Figure 3D). Hemodynamic performance of transradial BAV was unaffected in patients with bicuspid valves (Supplemental Table S7), and the overall performance remained similar after applying an alternative definition of hemodynamic success based on valve area for low-gradient AS (Supplemental Table S8). Moreover, the procedural time and contrast volume were significantly lower in the transradial group after adjustment, *P* < .001, 95% CI of –46.2 to –21.5 minutes for procedural time; and *P* = .004, 95% CI of –58.0 to –10.88 mL for contrast use (Table 2).

**Table 2:** Procedural characteristics and hemodynamic outcome

	Femoral (n = 148)	Radial (n = 105)	*P* value
Access			
Right radial	0	86 (81.9%)	<.001
Left radial	0	10 (9.5%)	–
Right ulnar	0	5 (4.8%)	–
Left ulnar	0	1 (1.0%)	–
Right femoral	134 (90.5%)	3 (2.9%)	–
Left femoral	14 (9.5%)	0	–
Anesthesia			
General anesthesia	2 (1.4%)	2 (1.9%)	.73
Conscious sedation	146 (98.6%)	103 (98.1%)	–
Concurrent procedures			
Coronary arteriogram	66.22%	66.67%	.94
PCI	18.92%	12.38%	.16
Right heart catheterization	66.22%	60.95%	.39
Access sheath size			<.001
8F	96 (65.3%)	95 (90.5%)	–
9F	12 (8.2%)	10 (9.5)	–
10F	18 (12.2%)	0	–
11F	1 (0.7%)	0	–
12F	2 (1.4%)	0	–
14F	15 (10.2%)	0	–
16F	3 (2.0%)	0	–
Balloon size			.12
16 mm	2 (1.4%)	0	–
18 mm	14 (9.5%)	10 (9.5%)	–
20 mm	42 (28.6%)	23 (21.9%)	–
22 mm	61 (41.5%)	65 (61.9%)	–
24 mm	22 (15.0%)	7 (6.7%)	–
25 mm	1 (0.7%)	0	–
26 mm	3 (2.0%)	0	–
28 mm	2 (1.4)	0	–
Balloon type			<.001
Tyshak (B. Braun Interventional Systems Inc)	65 (44.2%)	77 (73.3%)	–
Atlas (Becton Dickinson)	58 (39.5%)	22 (21.0%)	–
Vida (Bard)	9 (6.1%)	6 (5.7%)	–
True Dilatation (Becton Dickinson)	7 (4.8%)	0	–
True Flow (Becton Dickinson)	7 (4.8%)	0	–
Z-med (B. Braun Interventional Systems Inc)	1 (0.7%)	0	–
No. of balloon inflations			.12
1	57 (38.78%)	28 (27.18%)	–
2	57 (38.78%)	47 (45.63%)	–
3	27 (18.37%)	20 (19.42%)	–
4 or more	6 (4.08%)	7 (6.79%)	–
Rapid pacing method			<.001
None	11 (7.4%)	1 (1.0%)	–
Over-the-wire	48 (32.4%)	89 (84.8%)	–
Transvenous pacing	88 (59.5%)	15 (14.3%)	–
Preexisting pacemaker	1 (0.7%)	0 (0.0%)	–
Closure method			<.001
Pressure wrist band^a^	0	105 (100%)	–
Suture-based device^a^	118 (80.8%)	0	–
Collagen plug	15 (10.3%)	0	–
Secondary manual pressure	6 (4.1%)	0	–
Sheath kept in place	4 (2.7%)	0	–
Balloon rescue	2 (1.4%)	0	–
Covered stent	1 (0.7%)	0	–
Procedural efficiency			
Procedural time, min	98.70 ± 52.33	65.03 ± 26.71	.001
Fluoroscopy time, min	23.73 ± 19.02	17.39 ± 11.03	.003
Contrast volume, mL	65.15 ± 167.75	25.78 ± 28.65	.019
Radiation total air kerma, mGy	696.33 ± 858.01	537.25 ± 576.86	.10
Technical success	146 (98.6%)	101 (96.2%)	.21
Procedural challenges			
Sheath or balloon delivery failure	1 (0.7%)	4 (3.8%)	.07
Arterial dissection	1 (0.7%)	0	.40
Balloon rupture	4 (2.7%)	1 (1.0%)	.32
Balloon entrapment	0	1 (0.95%)	.23
Closure device failure	13 (9.0%)	2 (1.9%)	.022
Hemodynamic outcome			
Hemodynamic success	75.00%	78.10%	.57
Transcatheter Peak gradient reduction, mm Hg	14 (9-24)	16 (11-24)	.30
Mean echo gradient drop, mm Hg	8.93 ± 10.03	7.97 ± 10.33	.49
Increase in valve area, cm^2^	0.15 ± 0.40	0.17 ± 0.32	.73
Increased AI severity	20 (13.5%)	18 (17.1%)	.43

Values are n (%), mean ± SD, or median (IQR).

AI, aortic insufficiency; mGY, milligray; PCI, percutaneous coronary intervention.

a Closure method refers to the method that achieves hemostasis. All patients in the radial group had a pressure wrist band as the first option, and all patients in the transfemoral group had a suture-based device as the first option.

### Clinical outcome

The primary outcome occurred at a significantly higher rate in the transfemoral group compared to the transradial group: 17.47% of the transfemoral group and 2.53% in the transradial group (adjusted odds ratio, 0.13; 95% CI, 0.04-0.49; *P* = .003) (Central Illustration). Access-related events occurred in 9.4% of the femoral group and 0.92% of the radial group (resulting from a single case of balloon entrapment) (adjusted odds ratio, 0.10; 95% CI, 0.01-0.91; *P* = .041). Nonaccess-related events occurred in 7.17% of the transfemoral group and 1.61% of the transradial group (adjusted OR, 0.20; 95% CI, 0.04-0.88; *P* = .034). Periprocedural death rates were similar: 0.85% in the transfemoral group and 0.67% in the transradial group (*P* = .91) (Table 3). Multivariate logistic regression identified left ventricular over-the-wire pacing and lower diameter to LVOT diameter ratio as the mediators of the reduced nonaccess-related events in the transradial group (Supplemental Tables S5 and S6). Reexpressing periprocedural stroke as an access-related event as a sensitivity analysis did not impact the clinical outcome (Supplemental Table S4).

**Table 3:** IPTW-adjusted periprocedural and 30-day clinical outcomes.

Outcomes	Transfemoral (n = 269.30)	Transradial (n = 222.03)	Measure of association	*P* value
Periprocedural safety outcome			Odds ratio (95% CI)	
Primary composite	17.47%	2.53%	0.13 ( 0.04-0.49)	.003
Access-related events	9.40%	0.92%	0.10 (0.01-0.91)	.041
Modified major vascular	8.99%	0	–	–
Grade 3-4 bleeding	8.89%	0	–	–
Balloon entrapment	0	0.92%	–	–
Nonaccess-related events	7.17%	1.61%	0.20 (0.04-0.88)	.034
Hypotension	2.56%	1.14%	0.40 (0.08-2.12)	.28
Complete heart block	3.20%	0.47%	0.09 (0.05-1.56)	.098
Severe AI	0.90	0	–	–
Periprocedural stroke	0.51%	0	–	–
Periprocedural death^a^	0.85%	0.67%	0.87 (0.09-8.49)	.91
Other events				
Minor vascular	9.84%	5.82%	0.58 (0.19-1.79)	.35
Grade 1-2 bleeding	0.98%	0	–	–
Clinical events at 30 days^b^			Hazard ratio (95% CI)	
Secondary composite outcome	17.95%^b^	18.64%^b^	1.30 (0.56-3.03)	.54
Stroke	2.18%	1.00%		
Cardiac rehospitalization	2.14%	3.19%	0.21 (0.02-2.45)	.21
Heart failure rehospitalization	4.95%	2.01%	0.42 (0.10-1.70)	.22
Discharge failure	0.5%	0		
All-cause mortality	13.82%	16.64%	1.23 ( 0.15-9.52)	.84

AI, aortic insufficiency; IPTW, inverse probability of treatment weighting.

a Univariate analysis was performed instead of multivariate logistic regression owing to the limited number of events and model overfitting.

b Cumulative event rates for secondary outcomes are approximations owing to 3 cases of lost to follow-up.

**Figure 4:**
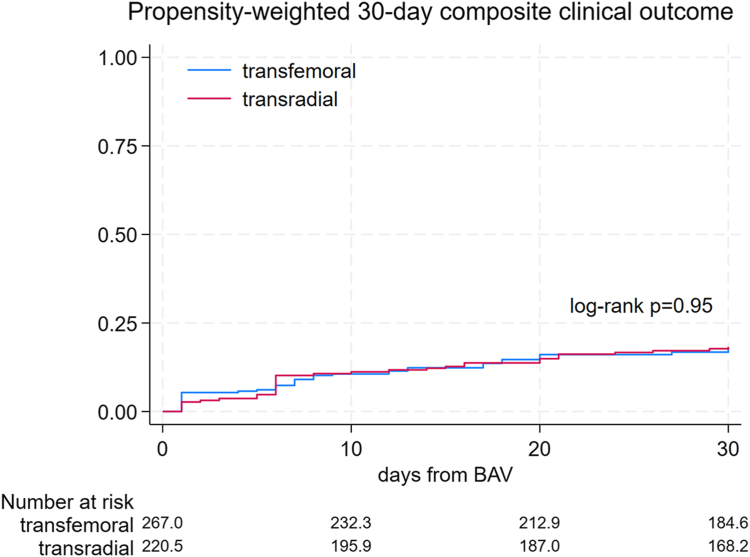
**Secondary outcome.** Inverse probability of treatment weighting–adjusted log-rank test and Kaplan-Meier estimates for the 30-day composite of all-cause mortality, heart failure rehospitalization, cardiac-related rehospitalization, and failure to discharge by day 30. BAV, balloon aortic valvuloplasty.

The secondary outcomes were similar in the transradial and transfemoral group (log-rank *P* = .95) (Figure 4) (hazard ratio, 1.30; 95% CI, 0.56-3.03; *P* = .54). Three patients out of 253 were lost to follow-up. Thirty-four patients (13.4%) underwent TAVR or surgical AV replacement and were censored.

No effect modification was found for the prespecified subgroups concerning the primary or secondary clinical outcomes (Supplemental Table S9). Results of the primary and secondary outcomes remained robust after multiple sensitivity analyses, with a significantly lower rate of primary outcome in the transradial group compared to the transfemoral group, and with no difference in the secondary outcome (Supplemental Tables S4-S6).

## Discussion

Our study is the first to objectively compare BAV via transradial and transfemoral access across multiple domains. The main findings of our study include favorable feasibility for radial access based on ultrasound except in patients with advanced kidney disease; similar hemodynamic performance between transradial and transfemoral BAV; shorter procedural times and lower contrast volumes with transradial access; a lower rate of periprocedural safety events for transradial BAV; and similar 30-day clinical outcomes in terms of all-cause mortality, rehospitalization, and discharge failure. Apart from 1 incident of balloon entrapment, there was no major complication.

An early feasibility study of radial access BAV from Japan using a 7F sheath and a different balloon platform has demonstrated the safety of transradial BAV but achieved only a low rate of hemodynamic success using a criterion based on change of AV area.^12^ Our study showed a high technical and hemodynamic success rate. Our femoral crossover rate is 3.8% which is similar to that reported in a European early feasibility study of 24 patients using more aggressive sheath and balloon sizing.^13^ We did not encounter any equipment delivery failure due to vessel tortuosity via the right radial approach as the preferred access. The Italian multicenter, single-arm Safety and Feasibility of Transradial Mini-Invasive Balloon Aortic Valvuloplasty (SOFTLY) I and II registries demonstrated the procedural feasibility and safety outcomes of radial access BAV regarding access-related complications and improvements in subsequent functional status in frail patients.^8^^,^^9^ SOFLTY II reported 0% VARC 2 major vascular event rate. However, there was 1 incident of balloon entrapment requiring surgical removal. Our study specifically included balloon entrapment as an access-related end point given the potential need for surgical bailout for this complication, and reassuringly still showed a significantly lower rate of access-related events with transradial access. Although the previously described technique in the limited literature did not require ultrasound guidance, we mandated 100% ultrasound guidance and the use of a shorter sheath to minimize the risk of vascular damage. This might improve case selection, procedure safety, and reproducibility. Other previously described techniques, such as biradial mini-BAV with peripheral balloons,^14^ were also performed at our center, but not included in this study.

The modest drop in the pressure gradient of 15 mm Hg (interquartile range 10 mm Hg, 24 mm Hg) is consistent with the results in recent literature.^4^^,^^8^^,^^9^ The management goal of modern BAV is to facilitate patient stabilization and stratification for further definitive treatment. There is no incentive to aggressively treat AS during the initial BAV procedure, especially because BAV alone produces neither long-term survival benefit nor sustained hemodynamic response. Balloons compatible with radial access are predominantly smaller-sized semicompliant balloons and might produce less pronounced hemodynamic effects compared to larger-bore noncompliant balloons. Reassuringly, we did not notice any compromise in the hemodynamic effect in patients with larger LVOT. The lack of difference in practice could be related to operator preference for smaller-profile, 8F compatible balloons, even in the transfemoral group, to minimize the risk of femoral access-related complications. A decrease in procedural time and contrast volume likely resulted from eliminating the time and contrast usage for femoral access, closure, and potential bailout, and the simplicity of over-the-wire pacing.

We are the first to study nonaccess-related events in transradial access BAV. The rationale behind this is the difference in mechanical properties of the balloons and sizing strategy, leading to potential differences in the risk of inducing valvular and perivalvular damage as well as disruption of atherosclerotic plaques upon retrieval of the balloons via the brachiocephalic or subclavian arteries, with the potential concern of stroke. We found a significantly lower rate of nonaccess-related events in the transradial group, mainly driven by a lower rate of complete heart block and hypotension, which remained valid after further multivariate regression adjusted for underlying bundle branch blocks and preexisting pacemakers. Multivariate logistic analysis showed that the lower rate in the radial group was mediated by the conservative balloon sizing and over-the-wire pacing. The conservative sizing of balloons might have resulted in less disruption of valvular calcification and subvalvular tissue. Indeed, deliberate undersizing of balloons has been used as a strategy to minimize the risk of adverse events in frail patients undergoing BAV.^15^ Our finding related to the use of over-the-wire pacing is potentially hypothesis-generating and warrants further investigation.

A few potential pitfalls related to the technique are worth mentioning. First, a backup sheath should always be available. In the case of balloon rupture, the sheath should be removed together with the balloon as a unit, followed by sheath replacement. Second, our technique utilizes a single radial access for simplicity, but in this method, continuous hemodynamic monitoring is disrupted during balloon delivery and inflation. In patients with a high likelihood of hemodynamic deterioration, such as low cardiac index and concomitant right heart disease along with multiple valvular regurgitations, contralateral radial access should be obtained for continuous invasive blood pressure monitoring, and other accesses should also be obtained for provisional mechanical circulatory support. Third, the sheath should be secured by suturing to the skin to avoid inadvertent movement, and an adequate dose of calcium channel blocker should be given via the access over the slender sheath prior to upsizing to avoid radial spasm.

### Study limitations and future directions

This single-center observational study is subject to limitations inherent to the study design. The baseline population was predominantly Caucasian men, which could potentially limit the generalizability of the study. Given the previously reported high patency rate of transradial BAV,^8^ we did not specifically assess radial patency in all patients. Although IPTW and multivariate regression were used to address confounders and multiple sensitivity analyses were performed to demonstrate the robustness of the finding, unmeasured confounders and selection bias could still occur. We defined stroke as a nonaccess-related event under the main analysis, but we understand that stroke could be secondary to valvular or artery-to-artery embolism or intracranial bleeding. The ultrasound criterion used to determine suitability is based on our previous clinical experience and the likelihood of passing 8F compatible equipment and was not based on published scientific data pertaining to this specific scenario. Future research should include multicenter registries, randomized controlled trials, and effectiveness analysis of ad hoc transradial BAV following diagnostic catheterization. Standardized training in case selection and procedural techniques is advisable for reproducing good procedural outcomes in lower-volume centers.

## Conclusion

Transradial access for BAV shows a significantly lower rate of periprocedural adverse events compared to transfemoral access while maintaining similar short-term clinical outcomes. This approach is feasible for most patients and provides comparable hemodynamic performance and success rates, along with a shorter procedural time compared to transfemoral BAV. In eligible patients, radial access may be preferred over femoral access.
