# Supplementary material for: Comparative Effectiveness of Balloon Aortic Valvuloplasty via Transradial and Transfemoral Access
Source: J Soc Cardiovasc Angiogr Interv. 2025 Nov 18;4(12):104015. doi: 10.1016/j.jscai.2025.104015 (PMC12766047; doi:10.1016/j.jscai.2025.104015)
Supplement: Supplementary Material [file mmc1.pdf]

Supplementary material

Table of Contents

<u>Supplementary Appendix S1</u>: A practical guide on the procedural technique for performing balloon aortic valvuloplasty through transradial access 2

Supplemental table S1. Balloons compatible with transradial BAV available in the United States 3

<u>Supplementary Appendix S2:</u> VARC III minor vascular events and grade I-II bleeding events 5

Supplementary table S2. Details of all access-related events: 5

Supplemental table S3. VARC III minor vascular complications according to access (radial vs ulnar) 5

<u>Supplementary Appendix S3:</u> Effect modification and sensitivity analysis 5

A. ***Effect modification on outcomes*** 6

Supplementary table S4. Test for effect modification on outcomes 6

B. ***Sensitivity analysis on outcomes*** 6

Supplementary table S5. Sensitivity analysis for primary and secondary outcome 6

Supplemental table S6. Additional multivariate logistic regression on predictors of non-access-related events. 7

Supplemental table S7. Balloon size-to-LVOT diameter ratio and over-the-wire pacing in transfemoral and transradial group 8

C. ***Sensitivity analysis on hemodynamic performance and procedural characteristics*** 8

Supplemental table S8. Additional analysis on hemodynamic performance according to subgroup (cardiogenic shock and ADHF) 8

Supplemental table S9. Additional analysis of hemodynamic performance according to valve subtype: trileaflet vs bicuspid 9

Supplemental table S10. Additional analysis on radial access usage according to AS type 9

Supplemental table S11. Additional analysis on hemodynamic success with alternative definition of hemodynamic success 10

Supplemental table S12. Additional analysis on pacing methods by AS type 10

<u>Supplementary Appendix S4:</u> Components of the propensity score and regression models 10

Supplementary table S13. Distribution of propensity score 11

Supplemental figure S1. Area under the receiver operating characteristics curve of the propensity score for the discrimination between transfemoral and transradial access usage 11

Supplemental figure S2. Propensity score balance kernel plot in raw and weighted population 12

Supplemental table S14. Multivariate regression models used following IPTW for further adjustments for confounders 13

<u>Supplementary Appendix S5:</u> Characteristics and outcomes in baseline population before IPTW 14

Supplemental table S15. Baseline characteristics before IPTW 14

Supplemental table S16. Detailed outcomes in baseline population and after IPTW 16

Supplementary Appendix S1: A practical guide on the procedural technique for performing balloon aortic valvuloplasty through transradial access

Ultrasound-Guided Access

The transradial BAV procedure was standardized with 100% ultrasound-guided access. First, both radial arteries are screened via ultrasound for characteristics including minimum diameter and any significant circumferential calcification. It is important to assess the length of the radial arteries from the elbow to the wrist before preparing the patient. In patients with significantly larger ulnar arteries than radial arteries, the use of the ulnar artery may be considered based on operator preference. An artery with a minimum diameter of 2.5 mm and without circumferential calcification is deemed suitable for access (Figure 2, Panel A).

Ultrasound-guided puncture is performed using a standard micropuncture access needle included in most modern radial access slender sheath sets. A single anterior puncture technique is preferred over a double puncture and drawback technique. Once access is obtained, a slender 6 French or 7-in-6 French slender radial sheath is placed over a 0.018-inch wire. The side port of the sheath is connected to pressure tracing, and an adequate dosage of calcium channel blocker, titrated against blood pressure, is administered to avoid vasospasm. Typically, a dosage of 200 micrograms of intra-arterial nicardipine is delivered through the sheath, with a low dose used in cases of borderline blood pressure. An intra-arterial or intravenous unfractionated heparin bolus is given to achieve a therapeutic activated clotting time (ACT) of at least 250 seconds. Nitrates should be avoided in the presence of aortic stenosis.

The access is then upsized to an 8 French short sheath (Merit Medical, Utah, USA) (Figure 2, Panel B) over a regular 0.035-inch J-tip wire or a stiff 0.035-inch wire when appropriate, followed by suturing the 8 French sheath to the skin (Figure 2, Panel C). We recommend starting with a 7-in-6 French slender sheath instead of a 6 French slender sheath to achieve smoother upsizing to the 8 French sheath. In cases where diagnostic cardiac catheterization is planned, the diagnostic procedure may be performed using the initial 6 or 7-in-6 sheath, followed by upsizing afterward upon completion of the diagnostic part of the procedure. It is important to suture the 8 French sheath to the skin to prevent inadvertent removal of the sheath during balloon retrieval.

A 9 French sheath can also be used in selected patients with a radial artery diameter of 3 mm or greater without circumferential calcification. One-third of the sheath length is wedged into the artery with the insertion of the entire sheath to prevent vascular injury. The use of a 9 French sheath permits the use of balloon sizes up to 24 mm.

Aortic Valve Crossing and Wire Placement

The aortic valve is crossed using a standard technique for aortic stenosis with a straight-tip wire and a catheter, such as an Amplatz Left 1 catheter. The straight-tip wire is then exchanged for a J-tip wire, allowing passive exposure of the wire in the left ventricle near the left ventricular outflow tract (LVOT). Following this, a dual-lumen pigtail catheter (Langston, Teleflex, USA) is delivered to the left ventricular outflow tract, with active withdrawal of the J-wire tip into the pigtail, forming the pigtail

shape near the LVOT and advancing the formed tip towards the left ventricle (LV) apex to measure the transaortic valve gradient. A circular-tip 0.035 wire is then actively delivered through the dual-lumen pigtail catheter to the LV apex. We prefer using the Confida wire (Medtronic, USA) due to its lower pacing threshold compared to other circular-tip wires. The use of manually shaped stiff wires should be avoided because of the risk of left ventricular perforation at the transition zone from the soft to stiff portion of the wire. Rapid pacing is performed using the over-the-wire pacing technique, with the anode connected via an alligator clamp to a needle tunneled under the skin (Figure 2, Panel C) and the cathode connected via an alligator clamp to the back of the circular-tip wire.

Balloon Selection and Delivery

Supplemental Table 1 lists the balloons we have tested that are compatible with the 8 French short sheath. These balloons are available in the United States. There are additional 8 French-compatible balloons available in other countries that are not listed here. For example, a 20mm balloon, which can be overstretched to 22mm, is available in Europe (VARCS II, Osypka, Rheinfelden, Germany), and a 7 French-compatible balloon is available in Japan (TRIVAL, Kaneka Medix, Japan). These sizes refer to cases where BAV is performed using a single balloon. An alternative technique that involves using two small balloons in parallel is beyond the scope of this article.

Supplemental table S1. Balloons compatible with transradial BAV available in the United States

Sheath Size	Balloon	Size
**8 French**	Tyshak II (B. Braun, USA)	18mm, 20mm, 22mm
	VIDA (BD, USA)	16mm, 18mm, 20mm
	Atlas Gold (BD, USA)	16mm, 18mm, 20mm
**9 French**	Tyshak II	24mm
	VIDA (BD, USA)	22mm
	Atlas Gold (BD, USA)	22mm

The radial compatibility shown above refers to new balloons only and not reused balloons. We advise operators not to readvance used balloons via the upper limb arterial system. When prepping the balloon, care should be taken to avoid inadvertent inflation before use. We size the balloon 1:1 to the left ventricular outflow tract (LVOT) diameter measured on the parasternal long-axis view on transthoracic echocardiography. If the measured LVOT diameter is larger than the maximum radial-compatible balloon size, the largest available balloon via the 8 French short sheath (22mm Tyshak) is used unless there is a compelling reason to use a 9 French sheath.

Access Closure

A pressure wristband is used for access closure. We recommend using wristbands with an inner rigid layer, such as the TR band (Terumo, USA), instead of pressure bands with only a soft outer layer, as those without a rigid inner layer may slide distally, resulting in closure failure and hematoma formation. The area around the wrist should be dried thoroughly before applying the band. The band is positioned with its marker at the arterial insertion point, typically 1 cm proximal to the skin puncture site. The balloon is partially inflated, followed by sheath removal.

A patent hemostasis protocol is preferred for radial arteries, while a fixed-pressure approach is recommended for ulnar arteries, especially when the artery is deep. This distinction relies entirely on operator experience regarding the increased risk of hematoma formation when using a patent hemostasis protocol for ulnar access. If achieving hemostasis proves difficult, the 0.035 wire may be kept in place during wristband inflation and sheath removal, followed by the removal of the wire and adjustment of the volume in the wristband once adequate hemostasis is ensured. Alternatively, in cases of suboptimal hemostasis, the 8 French sheath can be reinserted, and the pressure band can be repositioned or re-selected for the appropriate size of the wrist, repeating the process as necessary. We do not recommend using hydrophilic polymer sealing devices for this procedure.

Troubleshooting

Radial Spasm, Balloon Entrapment, and Rupture

Operators should be familiar with the standard management algorithm for radial artery spasm. If balloon rupture occurs, the sheath should be removed with the balloon as a unit over the 0.035-inch wire, followed by the insertion of a new sheath. Forceful pulling should be avoided to prevent retention of balloon fragments. In cases of balloon entrapment, femoral access and snaring may be necessary to free the balloon. If removal via alternative access is required, the balloon shaft must be cut. A backup sheath should always be available in the room.

Hemodynamic Monitoring and Mechanical Circulatory Support

Hypotension following balloon aortic valvuloplasty may occur, secondary to ventricular stunning and valvular regurgitation following rapid pacing or right ventricular failure. Rapid pacing runs should be kept as short as possible. Additional arterial access with continuous arterial monitoring during balloon inflation should be considered for high-risk patients. Most cases can be performed using a single access; however, arterial pressure monitoring is not possible during balloon inflation. In patients with a significantly decreased cardiac index or multivalvular regurgitation, especially with concomitant right ventricular dysfunction and tricuspid regurgitation, a backup axis for mechanical circulatory support should be prepared. Right heart catheterization should be performed to understand the hemodynamics of the patient prior to the balloon valvuloplasty procedure. The use of pulmonary arterial catheters via jugular access should also be considered to monitor pulmonary arterial pressure. If right ventricular support is needed, the pulmonary arterial catheter can be switched to a right ventricular support microaxial flow pump via a stiff wire, followed by upsizing venous access. If a high likelihood of left ventricular support is anticipated, then the procedure should be performed via transfemoral access instead of transradial access.

Hematoma

Forearm hematomas typically occur just proximal to the pressure band after closure and are caused by an overly distal band position or movement of the pressure band after placement. If this occurs, another pressure band placed proximal to the first band can mitigate the problem. Preprocedural screening of the radial artery up to the elbow is necessary to prevent hematoma development from vascular injury in the proximal radial artery segment. Radial loops should be approached meticulously during the initial wire passing. Most forearm hematomas can be managed conservatively with cyclical blood pressure cuff inflation, pressure bandages, and arm elevation.

Supplementary Appendix S2: VARC III minor vascular events and grade I-II bleeding events

Supplementary table S2. Details of all access-related events:

	Transradial (N=105)	Transfemoral (N=148)
**VARC III minor vascular and grade I-II bleeding**	(8)	(12)
	Closure device failure requiring two or more TR bands(2)	Closure device failure (7)
	EASY I-II hematoma (4)	Groin hematoma (3)
	Pseudoaneurysm (2)	Iliac dissection (1)
		Hemoglobin drop requiring transfusion (1)
**Modified VARC III major vascular and grade 3-4 bleeding**	(1)	(10)
	Balloon entrapment requiring removal from femoral access (1)	Iliac artery laceration and hypotension (1)
		Access site bleeding with hypotension (4)
		Fatal intracranial bleeding (1)
		Embolic event requiring below knee amputation (1)
		Retroperitoneal hemorrhage (3)

Supplemental table S3. VARC III minor vascular complications according to access (radial vs ulnar)

	radial<br>n=96	ulnar<br>n=6	
VARC III minor<br>Vascular complications	6 (6.2%)	2 (33.3%)	p=0.017

Finding: There was a significantly higher rate of minor vascular complications associated with the use of ulnar access, as 2 out of 6 cases developed hematomas, both of which were managed conservatively.

Supplementary Appendix S3: Effect modification and sensitivity analysis

Effect modification was not observed for concurrent PCI, peripheral artery disease, chronic kidney disease, or cardiogenic shock, and acute decompensated heart failure (ADHF) concerning the periprocedural or 30-day outcomes (Supplementary Table 2). All p-for-interaction values exceeded 0.05. The results for the primary and secondary outcomes remained consistent after several sensitivity analyses, showing significantly lower periprocedural event rates in the radial group and similar event rates for the 30-day clinical outcome (Supplementary Table 4).

A. Effect modification on outcomes

Supplementary table S4. Test for effect modification on outcomes

Primary outcome		Odds ratio		P for interaction
	PCI			Omitted
	No PCI			
	PAD			Omitted
	No PAD			
	CKD		0.48 (0.12-1.86)	0.181
	No CKD		0.88 (0.01-0.71)	
	Cardiogenic shock		0.02 (0.01-0.84)	0.198
	No Cardiogenic shock		0.18 (0.04-0.80)	
	ADHF	0.07 (0.01-0.44)		0.372
	No ADHF	0.21 (0.02-2.60)		
Secondary outcome		Hazard ratio		
	PCI	1.32 (0.32-5.56)		0.447
	No PCI	0.71 (0.39-1.28)		
	PAD	0.39 (0.13-1.19)		0.156
	No PAD	1.02 (0.53-1.95)		
	CKD	0.82 (0.42-1.63)		0.871
	No CKD	0.76 (0.29-1.95)		
	Cardiogenic shock	0.93 (0.30-2.89)		0.579
	No Cardiogenic shock	0.68 (0.36-1.29)		
	ADHF	1.30 (0.54-3.12)		0.062
	No ADHF	0.22 (0.05-1.09)		

B. Sensitivity analysis on outcomes

Multiple sensitivity analyses were performed, with overall agreement in the results indicating a significantly lower rate of primary outcomes in the transradial group compared to the transfemoral group, and similar secondary outcomes in both the transradial and transfemoral groups.

Supplementary table S5. Sensitivity analysis for primary and secondary outcome

Method	outcome	Adjusted MOA (95% CI)	p
IPW trimmed to 99 percentile	Primary	OR 0.19 (0.05-0.71)	0.014
	Secondary	HR 0.58 (0.40-1.92)	0.75
Propensity-score matched*	Primary	** no event in radial group	<0.001
	Secondary	HR 1.17 (0.31-4.36)	0.814
All variates in regression model	Primary	OR 0.003 (0.00009-0.16)	0.004
	secondary	Proportional hazard Assumption violated	
Concurrent PCI excluded	Primary	OR 0.26(0.06-1.05)	0.06
	Secondary	HR 0.68 (0.33-1.42)	0.31
PAD excluded	primary	OR 0.20 (0.05-0.82)	0.026
	secondary	HR 1.03 (0.46-2.31)	0.94
CKD excluded	Primary	OR 0.02 (0.002-0.34)	0.006
	secondary	HR 0.77 (0.24-2.40)	0.65

Adjust by operator	Primary	OR 0.06 (0.01-0.28)	<0.001
	secondary	HR 0.70 (0.31-1.58)	0.39
Adjusting for time to discharge	secondary	HR 0.85 (0.28-2.59)	0.773
Redefining non-cardiac death as Compete event in 30-day outcome**	secondary	SHR 0.95 (0.38-2.42)	0.92
Redefining stroke as an access-related event	Primary	OR 0.13 (0.35-0.48)	0.002
Access related events		OR 0.087, (0.01-0.84)	0.035
Non-access related events		OR 0.23 (0.06-0.95)	0.042

MOA: measures of association.
*Greedy algorithm with caliper of 0.2 of SD of the logit of the propensity score was used for propensity score matching
OD: odds ratio, HR hazard ratio, SHR: subdistribution hazard ratio
**. The Fine and Gray method with competing risk regression model was used to obtain the subdistribution hazard ratio.

Supplemental table S6. Additional multivariate logistic regression on predictors of non-access-related events

Non-access-related events	Odds ratio	p-value	95% confidence	interval
balloon size to LVOT diameter ratio*	2.72	0.024	1.14	6.50
Over-the-wire pacing	0.21	0.016	0.06	0.74
Semi-compliant balloon	1.26	0.785	0.24	6.65
Radial access	0.07	0.135	0.00	2.31
RBBB	3.54	0.183	0.55	22.93
Atrial fibrillation	0.18	0.21	0.01	2.68
eGFR	1.03	0.149	0.99	1.07
PAD	4.28	0.185	0.50	36.94
Pre-existing pacemaker	208.59	0.102	0.34	127297.50
ADHF	0.37	0.48	0.02	5.98
Cardiogenic shock	12.14	0.137	0.45	328.41
Valve type (compared to trileaflet)				
Bicuspid valve	3.38	0.342	0.27	41.99
bioprosthetic valve	3.83	0.25	0.39	37.86
AS subtype (compared to high-gradient)				
LFLG	0.04	0.203	0.00	5.67
Paradoxical LFLG	5.22	0.232	0.34	79.20
NFLG	0.16	0.25	0.01	3.75
Number of balloon inflations	1.21	0.643	0.53	2.75
LVEF (%)	0.99	0.865	0.93	1.06
Prior stroke	0.39	0.373	0.05	3.07
Mitral valve disease	1.51	0.674	0.22	10.41
Tricuspid valve disease	0.49	0.525	0.05	4.53
RV dysfunction	2.61	0.649	0.04	165.52

*Balloon size to LVOT diameter ratio was expressed as the balloon diameter divided by the LVOT diameter measured on echocardiography times 10. Since the value was multiplied by 10, the odds ratio of 2.72 refers to every increase of 0.1 unit of increase in balloon diameter relative to LVOT diameter (instead of every 1 unit increase in the ratio of the diameters.)

In the main analysis, we found an unexpectedly low rate of non-access-related events in the transradial group compared to the transfemoral group. We conducted additional analysis to explain this finding. The balloon size relative to the LVOT diameter and the use of over-the-wire pacing were identified as independent predictors of non-access-related events. These two variables acted as mediators for the lower occurrence of non-access-related events in the transradial group, as the rate of over-the-wire pacing was significantly higher in that group, and the balloon diameter to LVOT diameter ratio was notably lower. It is important to note that these two are not confounders but mediators, as the use of radial access limited the available balloon sizes, resulting in the relative undersizing of balloons. The use of radial access also influenced the pacing method by leading to an operator preference for over-the-wire pacing instead of obtaining additional venous access for temporary pacing in order to streamline the procedure.

Supplemental table S7. Balloon size-to-LVOT diameter ratio and over-the-wire pacing in transfemoral and transradial group

	N=148 Transfemoral	N=105 transradial	p
Balloon Sizing ratio (x 10)	10.29 1.08	10.01 0.95	0.037
Over-the-wire pacing	48 (32.4%)	89 (84.8%)	<0.001

C. Sensitivity analysis on hemodynamic performance and procedural characteristics

Supplemental table S8. Additional analysis on hemodynamic performance according to subgroup (cardiogenic shock and ADHF)

	TFV n=133	TRV n=91		Subgroup No cardiogenic shock
Mean gradient reduction (mmHg)	8.97 ± 10.04	7.67 ± 10.17	p = 0.37	
Contrast volume	68.52 ± 176.46	24.26 ± 26.58	p = 0.019	
Radiation (air kerma, mGy)	710.33 ± 893.57	525.87 ± 574.79	p = 0.09	
Procedural time (min)	99.41 ± 52.60	64.49 ± 25.46	p < 0.001	
	TFV n=15	TRV n=14		cardiogenic shock
Mean gradient reduction (mmHg)	8.56 ± 10.31	10.21 ± 11.72	p = 0.71	

Contrast volume	35.53 ± 32.46	35.57 ± 39.32	p = 1.00	
radiation (air kerma. mGy)	573.20 ± 433.57	610.43 ± 606.69	p = 0.85	
Procedural time (min)	92.60 ± 51.35	68.43 ± 34.56	p = 0.15	
	TFV<br>n=57	TRV<br>n=31		No ADHF
Mean gradient reduction (mmHg)	8.15 ± 10.31	6.88 ± 7.71	p = 0.60	
Contrast volume	56.42 ± 52.01	27.42 ± 32.93	p = 0.006	
radiation (air kerma, mGy)	698.26 ± 841.08	469.26 ± 474.97	p = 0.17	
Procedural time (min)	97.57 ± 51.01	70.29 ± 31.21	p = 0.008	
	TFV<br>n=91	TRV<br>n=74		ADHF
Mean gradient reduction (mmHg)	9.44 ± 9.88	8.32 ± 11.06	p = 0.52	
Contrast volume	70.69 ± 210.67	25.08 ± 26.84	p = 0.07	
radiation (air kerma, mGy)	695.11 ± 873.25	566.12 ± 615.80	p = 0.29	
Procedural time (min)	99.42 ± 53.43	62.76 ± 24.41	p < 0.001	

TFV: transfemoral valvuloplasty. TRV: transradial valvuloplasty

Supplemental table S9. Additional analysis of hemodynamic performance according to valve subtype: trileaflet vs bicuspid

Drop in mean gradient (mmHg)	Valve type<br>trileaflet	bicuspid	p-value
	N=93	N=10	
Transadial	6.25 (1-12)	6.55 (3.6-12.49)	0.98
	N=129	N=12	
transfemoral	7.8 (3.3-13.75)	3.2 (.4-11.5)	0.16

Supplemental table S10. Additional analysis on radial access usage according to AS type

	High-gradient	LFLG	Paradoxical LFLG	NFLG	
Radial access usage	39.6%	37.7%	51.9%	37.5%	P=0.37

LFLG: low-flow low-gradient. NFLG: normal-flow low gradient

Supplemental table S11. Additional analysis on hemodynamic success with alternative definition of hemodynamic success

Hemodynamic success	Radial	Femoral	P value
High-gradient	78.6% (33/42)	76.6% (49/64)	0.81
LFLG	52.2% (12/23)	44.7% (17/38)	0.57
Paradoxical LFLG	53.6% (9/26)	34.6% (15/28)	0.16
NFLG	16.7% (2/12)	45.0% (9/20)	0.10

Owing to concerns that change in pressure gradient may not be a reliable indicator of hemodynamic success in patients with low-gradient AS, additional analysis was performed with altered definition of hemodynamic success. For high gradient AS, a drop in 30% or more in pressure gradient was used. In low-flow low-gradient (LFLG) and paradoxical LFLG AS, an increase of valve area by 30% or more or an increase of valve area to 1cm^2 or more was used. In normal-flow low-gradient (NFLG) gradient, both criteria for a drop in gradient by 30% and an increase in area by more than 30% had to be satisfied. Using these alternative definitions, the success rate in the low-gradient groups was found to be lower. The analysis was underpowered. Nevertheless, the result was overall comparable between transradial and transfemoral groups.

Supplemental table S12. Additional analysis on pacing methods by AS type

	High gradient	LFLG	Paradoxical LFLG	NFLG	
Rapid pacing method	n=106	n=61	n=54	n=32	P=0.05
None (Trueflow balloon)	2 (1.9%)	7 (11.5%)	2 (3.7%)	1 (3.1%)	
Over-the-wire LV pacing	57 (53.8%)	30 (49.2%)	36 (66.7%)	14(43.8%)	
RV temporary pacing wire	47 (44.3%)	23 (37.7%)	16 (29.6%)	17(53.1%)	
Pre-existing permanent pacemaker	0 (0.0%)	1 (1.6%)	0 (0.0%)	0 (0.0%)	

Supplementary Appendix S4: Components of the propensity score and regression models

43 variables were included in the propensity score, including the following:

Demographics variables

male sex, body height, body weight, race, age, hypertension, hyperlipidemia, diabetes, cigarette smoking, heart failure, atrial fibrillation, coronary artery disease, chronic kidney disease, dialysis , peripheral artery disease, chronic obstructive pulmonary disease or other lung disease, preexisting pacemaker, prior or active malignancy, prior acute coronary syndrome, prior stroke, prior percutaneous coronary intervention, prior coronary artery bypass-grafting, pre-existing bundle branch block, emergency admission, acute decompensated heart failure, cardiogenic shock,

anatomy and laboratory result variables

Aortic stenosis subtype, valve morphology, Concomitant mitral valve disease, concomitant tricuspid valve disease, Left ventricular ejection fraction (LVEF), Hemoglobin, creatinine, estimated glomerular filtration rate (eGFR),

Procedural characteristics and medications variables
Aspirin, clopidogrel, Direct oral anticoagulant (DOAC), warfarin
concomitant coronary arteriogram, concomitant PCI, month and year of BAV, type of anesthesia

Supplementary table S13. Distribution of propensity score

	femoral	Radial
Minimum value	.003915	.0835916
P1	.0048199	.0957349
P5	.0113914	.1512731
P10	.0175782	.296683
P25	.0657716	.4486335
P50	.1838745	.6906701
P75	.3509671	.8715345
P90	.6045982	.9563811
P95	.7902789	.9711741
P99	.9315441	.9849187
Maximum value	.9469652	.9881428

Supplemental figure S1. Area under the receiver operating characteristics curve of the propensity score for the discrimination between transfemoral and transradial access usage

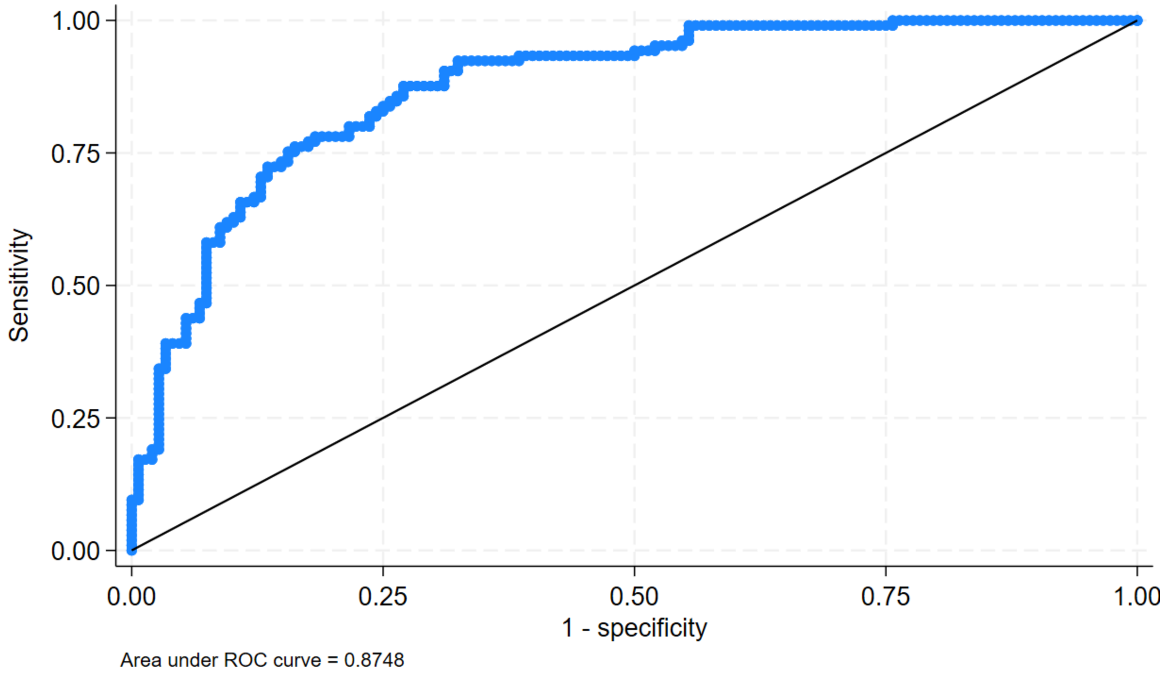

Discrimination: Area under the ROC curve = 0.9125

Information criteria: Akaike 310.5108 Bayesian 462.465

Goodness of fit: hosmer-Lemeshow p=0.35

An alternative propensity with AUROC of 0.9125 with the addition of rapid pacing methods, operator, surgical risk, and BAV indication was also created. This alternative propensity score was not used as it resulted in extreme outliers in inverse probability weight. Similarly, propensity scores creating that contained STS mortality percentages and frailty indexes using multiple imputation also led to extreme weights in some patients and were not used. Nevertheless, the overall result was consistent with significant difference in primary outcome and no difference in secondary outcome when using these propensity scores

Supplemental figure S2. Propensity score balance kernel plot in raw and weighted population

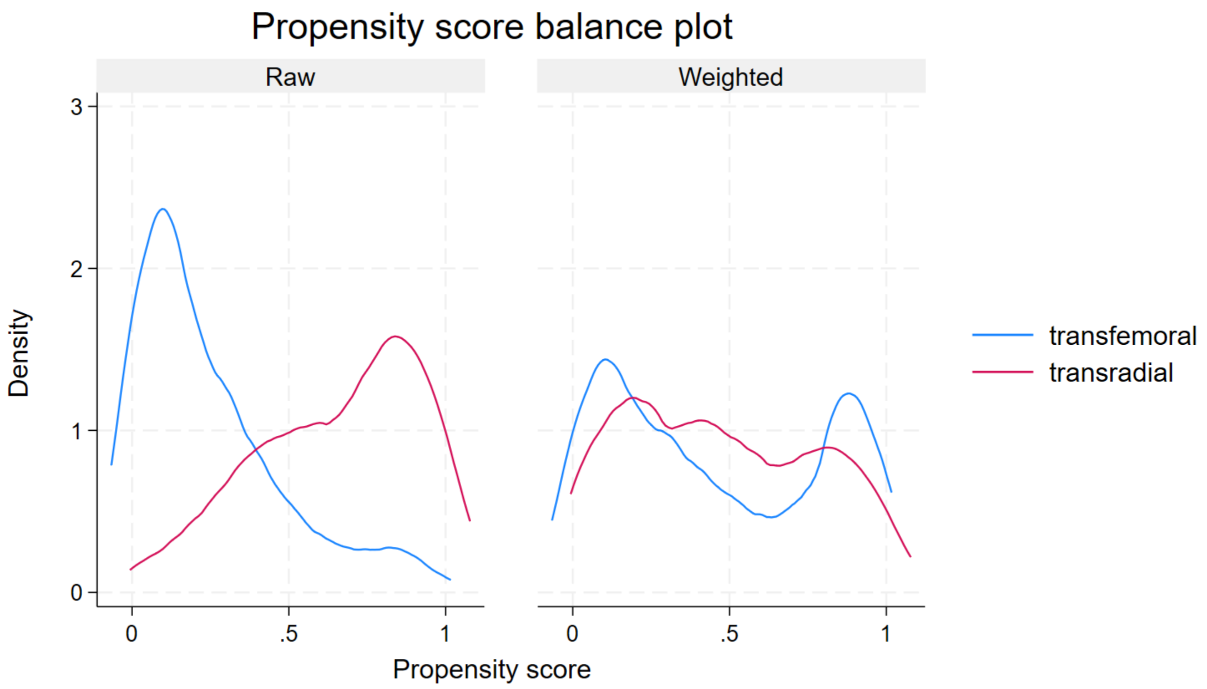

The propensity score was well balanced after inverse probability of treatment weighting, as shown in the kernel density plot:

Weighted multivariate regression models applied following IPTW

The following multivariate regression models were used for further adjustment of covariates following IPTW owing to the following variables having a standardized mean difference of ≥0.2 after IPTW. The logistic regression model was simpler owing to the limited observations which led to a decrease in the total number of subjects in the model when a more complex model including subgrouping variables were used.

Supplemental table S14. Multivariate regression models used following IPTW for further adjustments for confounders

Primary outcome: weighted logistic regression.

Primary outcome	Odds ratio	Standard error (linearized)	T statistic	p-value	95% confidence interval	
Radial access	0.13	0.09	-3.04	0.003	0.04	0.49
Nonwhite ethnicity	0.33	0.31	-1.18	0.241	0.05	2.12
AI severity	1.32	0.40	0.91	0.366	0.72	2.41
GFR	1.01	0.01	0.82	0.414	0.99	1.03
ECG pattern	0.89	0.30	-0.34	0.737	0.46	1.73
Indication	1.05	0.26	0.19	0.847	0.65	1.69
_constant	0.13	0.07	-3.64	0	0.04	0.39

Secondary outcome: weighted cox regression

Secondary outcome	Hazard Ratio	standard error (robust)	Z statistic	p value	95% confidence interval	
Radial access	1.30	0.56	0.62	0.538	0.56	3.03
Race						
African American	0.40	0.19	-1.92	0.054	0.16	1.02
Hispanic	0.67	0.31	-0.86	0.392	0.27	1.67
South Asian	2.47	2.19	1.02	0.309	0.43	14.05
East Asian	3.48	2.18	1.99	0.047	1.02	11.91
West Asian	4.97E-21	.	.	.	.	.
Others	1.20E-19	.	.	.	.	.
AI severity	1.37	0.30	1.45	0.148	0.89	2.10
GFR	0.99	0.01	-2.29	0.022	0.97	1.00
ECG						
LBBB	1.22	0.94	0.26	0.797	0.27	5.56
RBBB	1.04	0.50	0.07	0.941	0.40	2.66
pacing rhythm	0.32	0.24	-1.49	0.137	0.07	1.44
Indication						
Palliative	13.28	15.71	2.19	0.029	1.31	135.03
Stratifying	4.93	2.98	2.64	0.008	1.51	16.10
Urgent treatment	6.77	2.68	4.82	<0.001	3.11	14.72

Supplementary Appendix S5: Characteristics and outcomes in baseline population before IPTW

Supplemental table S15. Baseline characteristics before IPTW

	baseline femoral (N=148)	baseline radial (N=105)	SMD Baseline
**Demographics**			
age	78.89 ± 10.13	78.29 ± 10.03	0.06
male	46.62%	62.86%	-0.33
height	1.66 ± 0.12	1.68 ± 0.10	-0.22
Body weight	79.53 ± 23.64	81.90 ± 23.26	-0.10
Race			
Caucasian	77.71%	80.00%	0.06
African American	12.16%	12.38%	0.01
Hispanic	5.41%	4.76%	-0.03
South Asian	3.38%	0.00%	-0.26
East Asian	0.68%	0.00%	-0.12
West Asian	0.00%	2.86%	0.24
Other	0.68%	0.00%	-0.12
Hypertension	86.49%	91.43%	-0.16
hyperlipidemia	62.84%	69.52%	-0.14
Diabetes	52.03%	39.05%	0.26
Smoker	26.35%	54.29%	-0.59
Heart failure	45.95%	66.67%	-0.43
Atrial fibrillation	32.43%	44.76%	-0.26
Mitral valve disease	48.65%	31.43%	-0.35
Tricuspid valve disease	42.57%	43.81%	0.02
CAD	56.76%	55.24%	0.03
CKD	56.76%	52.38%	0.09
dialysis	18.92%	10.48%	0.24
PAD	25.68%	22.86%	0.07
COPD / lung disease	29.73%	25.71%	0.09
Pacemaker/ICD	9.46%	12.38%	-0.09
Malignancy	18.24%	11.43%	0.19
Dementia	2.70%	4.76%	0.02
GFR	54.45 ± 31.36	64.91 ± 29.53	-0.34
Hemoglobin	10.57 ± 2.01	10.92 ± 2.27	-0.16
Creatinine	2.19 ± 2.37	1.67 ± 1.78	0.25
Prior ACS	10.81%	21.90%	-0.30
Prior stroke	8.11%	12.38%	-0.14
Prior PCI	21.62%	27.62%	-0.14
Prior CABG	10.14%	9.52%	0.02
Emergency admission	56.76%	70.48%	-0.29
ADHF	61.49%	70.48%	-0.19
Cardiogenic shock	10.14%	13.33%	-0.10
STS mortality score	11.02 ± 12.92	7.86 ± 6.33	-0.31
Frailty index	1.54 ± 0.89	1.58 ± 0.93	0.04
KCCQ score	34.45 ± 22.41	34.10 ± 21.20	-0.02
Electrocardiogram			

normal QRS	68.24%	61.90%	-0.13
LBBB	9.46%	10.48%	0.03
RBBB	13.51%	18.10%	0.13
pacing			0.03
aspirin	68.24%	53.33%	0.31
Clopidogrel	27.70%	11.43%	0.42
DOAC	27.03%	22.86%	0.10
warfarin	3.38%	5.71%	-0.11
**Anatomical characteristics**			
LVEF	50.01 ± 15.58	51.22 ± 17.09	-0.07
AV mean gradient	32.33 ± 15.45	32.21 ± 14.37	-0.01
AV area	0.81 ± 0.35	0.78 ± 0.22	-0.11
AI severity			
none/trivial	57.14%	49.50%	0.07
mild	32.14%	35.64%	0.07
moderate	9.29%	12.87%	0.11
severe	1.43%	1.98%	0.04
Valve type			
Trileaflet	87.16%	88.57%	0.04
Bicuspid	8.11%	9.52%	0.05
prosthetic	4.73%	1.90%	-0.16
AS type			
High gradient	43.24%	40.00%	-0.07
low-flow low-gradient	25.68%	21.90%	-0.09
paradoxical low-flow low-gradient	17.57%	26.67%	0.22
normal flow low gradient	13.51%	11.43%	-0.06
LV end diastolic presssure	20.94 ± 9.95	20.05 ± 9.70	0.09
LVOT diameter	2.04 ± 0.36	2.14 ± 0.18	-0.34
RV dysfunction	22.39%	22.92%	0.01
**Adjusted Procedural characteristics**			
BAV month and year	202260.7 ± 84.27	202293.1 ± 89.63	0.37
BAV indication			
Bridge to AVR	68.92%	68.57%	-0.09
Palliative	2.03%	0.95%	-0.09
Stratifying	10.14%	13.33%	0.10
Urgent treatment	18.92%	17.14%	-0.05
General anesthesia			0.04
Concurrent angiogram	66.22%	66.67%	-0.01
Concurrent PCI	18.92%	12.38%	0.18

SMD: Standardized mean difference. CAD: coronary artery disease. CKD: Chronic kidney disease. PAD: peripheral artery disease. COPD: chronic obstructive pulmonary disease. GFR: glomerular filtration Rate. ACS: Acute coronary syndrome. PCI: Percutaneous coronary intervention. CABG: Coronary artery-bypass grafting. ADHF: Acute decompensated heart failure. STS: Society of Thoracic Surgeons. KCCQ: Kansas City Cardiomyopathy Questionnaire. QRS: QRS complex on electrocardiogram. LBBB: left bundle branch block. RBBB: Right bundle branch block. DOAC: direct oral anticoagulant. LVEF: left ventricular ejection fraction. AV: aortic valve. AI: aortic insufficiency. LV: left ventricular. LVOT: left ventricular outflow tract. RV: right ventricular.

Supplemental table S16. Detailed outcomes in baseline population and after IPTW

	Baseline TF N=148	Baseline TR N=105	MOA	p	IPTW TF N=269.30	IPTW N=222.03	Adj MOA (95% CI)	p
**Periprocedural safety outcome**	21 (14.19%)	4 (3.81%)	0.24	0.0064			Odds ratio(95% CI)	
**Primary composite**					17.47%	2.53%	0.13 (0.04 -0.49)	0.003
Access-related events	10 (6.76%)	1 (0.95%)		0.026	9.40%	0.92%	0.10 (0.01-0.91)	0.041
VARC-III major vascular	9 (6.1%)	0		0.01	8.99%	0		
VARC-III Grade 3-4 bleeding	9 (6.1%)	0		0.01	8.89%	0		
Balloon entrapment	0	1		0.23	0	0.092%		
Non-access-related events	11 (7.43%)	3 (2.86%)	0.37	0.12	7.17%	1.61%	0.20 (0.04-0.88)	0.034
Hypotension	4 (2.7%)	2 (1.9%)	0.70 (0.06-4.99)	0.68	2.56%	1.14%	0.40 (0.08-2.12)	0.28
Complete heart block	4 (2.7%)	1 (1.0%)	0.35	0.32	3.20%	0.47%	0.09 (0.05-1.56)	0.098
Severe AI	2 (1.4%)	0		0.23	0.90	0		
Periprocedural stroke**	1 (0.7%)	0		0.40	0.51%	0		
Periprocedural death*	2 (1.4%)	1 (1.0%)	0.70	0.77	0.85%	0.67%	0.87 (0.09-8.49)	0.91
**Other events**								
VARC-III Minor vascular	12 (8.1%)	8 (7.6%)	0.93	0.89	9.84%	5.82%	0.58 (0.19-1.79)	0.35
VARC-III Grade 1-2 bleeding	2 (1.4%)	0		0.23	0.98%	0		
**Clinical events at 30 days***							Hazard ratio (95% CI)	
Secondary composite outcome	37(25.00%)	21 (20.0%)	0.75 (0.43-1.30)	0.30	17.95%	18.64%	1.30 (0.56-3.03)	0.54
Stroke	5 (3.4%)	1 (1.0%)	0.35	0.32	2.18%	1.00%	***	
Cardiac rehospitalization	5 (3.4%)	5 (4.8%)	1.40 (0.41-4.83)	0.59	2.14%	3.19%	0.21 (0.02 – 2.45)	0.21
Heart failure rehospitalization	11 (7.4%)	3 (2.9%)	0.37 (0.10-1.32)	0.11	4.95%	2.01%	0.42 (0.10-1.70)	0.22
Discharge failure	1 (0.68%)	0		0.40	0.5%	0		
All-cause mortality	20 (13.5%)	17 (16.2%)	1.24 (0.63-2.44)	0.52	17.28%	19.55%	1.39 (0.16 – 10.55)	0.80

*Cumulative incidence for 30-day events are only for approximation as there were 3 cases of lost-to-follow up. The adjusted hazard ratio and p-values were used to assess the secondary outcomes.
**Periprocedural stroke and periprocedural death were defined as stroke or all-cause mortality within 24 hours of the procedure.
***proportional hazard assumption violated. Univariate model used
TF: Transfemoral. TR: Transradial. IPTW: inverse probability of treatment weighting. Adj MOA: Adjusted measure of association. MOA: Measure of association. AI: Aortic insufficiency.
